# Upper-Limb Kinematic Behavior and Performance Fatigability of Elderly Participants Performing an Isometric Task: A Quasi-Experimental Study

**DOI:** 10.3390/bioengineering10050526

**Published:** 2023-04-26

**Authors:** Helena Silva-Migueis, Eva María Martínez-Jiménez, Israel Casado-Hernández, Adriano Dias, Ana Júlia Monteiro, Rodrigo B. Martins, João Marcos Bernardes, Daniel López-López, Juan Gómez-Salgado

**Affiliations:** 1 Research, Health and Podiatry Group, Department of Health Sciences, Faculty of Nursing and Podiatry, Industrial Campus of Ferrol, Universidade da Coruña, 15403 Ferrol, Spain; h.smigueis@udc.es (H.S.-M.); ana.demacedo@udc.es (A.J.M.); daniel.lopez.lopez@udc.es (D.L.-L.); 2 Physiotherapy Department, Escola Superior de Saúde da Cruz Vermelha Portuguesa-Lisboa, 1300-125 Lisbon, Portugal; rmartins@esscvp.eu; 3 Facultad de Enfermería, Fisioterapia y Podología, Universidad Complutense de Madrid, 28040 Madrid, Spain; evamam03@ucm.es; 4 Department of Public Health, Graduate Program in Collective/Public Health, Botucatu Medical School, Universidade Estadual Paulista/UNESP, Botucatu 18610-307, SP, Brazil; dias.adriano@unesp.br (A.D.);; 5 Departamento de Sociología, Trabajo Social y Salud Pública, Universidad de Huelva, 21004 Huelva, Spain; salgado@uhu.es; 6 Safety and Health Postgraduate Programme, Universidad Espíritu Santo, Guayaquil 092301, Ecuador

**Keywords:** isometric activity, injury, musculoskeletal disorders, functional performance, wearable technologies, upper-limb movement

## Abstract

**Simple Summary:**

The purpose of this research was to understand how upper-limb kinetics behaves alongside activity time during a position-sustained isometric task and how upper-limb kinetics relates to performance fatigability. As hypothesized, there were changes in acceleration behavior indicative of the displacement of the upper limb in the direction of shoulder extension, especially in the second half of the task, and also an increasing variation of acceleration, and, thus, in movement variability, alongside activity time. However, these changes showed different behaviors between men and women, suggesting greater performance fatigability in women. Results also showed that performance fatigability was positively related to average acceleration in an early phase of activity only in men, meaning that early movement adjustments were apparently sufficient to increase activity time and, consequently, performance. According to the results of our study, upper-limb acceleration measured through a single IMU can be a useful and easy strategy to identify fatigue early.

**Abstract:**

Upper-limb position-sustained tasks (ULPSIT) are involved in several activities of daily living and are associated with high metabolic and ventilatory demand and fatigue. In older people, this can be critical to the performance of daily living activities, even in the absence of a disability. Objectives: To understand the ULPSIT effects on upper-limb (UL) kinetics and performance fatigability in the elderly. Methods: Thirty-one (31) elderly participants (72.61 ± 5.23 years) performed an ULPSIT. The UL average acceleration (AA) and performance fatigability were measured using an inertial measurement unit (IMU) and time-to-task failure (TTF). Results: The findings showed significant changes in AA in the X- and Z-axes (*p* < 0.05). AA differences in women started earlier in the baseline cutoff in the X-axis, and in men, started earlier between cutoffs in the Z-axis. TTF was positively related to AA in men until 60% TTF. Conclusions: ULPSIT produced changes in AA behavior, indicative of movement of the UL in the sagittal plane. AA behavior is sex related and suggests higher performance fatigability in women. Performance fatigability was positively related to AA only in men, where movement adjustments occurred in an early phase, though with increased activity time.

## 1. Introduction

Isometric activity is often used as an important tool for recreation, rehabilitation plans, and sports, to upgrade fitness, health, and functional efficiency, especially in the elderly, since it has beneficial effects on improving joint balance (free of joint movement) and decreasing blood pressure [1,2] and general pain [3].

Nevertheless, during more demanding isometric tasks, such as position-sustained tasks, in psychophysiological settings, rate-limiting actions occur, since the load adjustment needs for sustaining the limb are greater than for other kinds of assignments [4] and result in high metabolic and ventilatory demand and fatigue [5,6]

In upper-limb position-sustained tasks, such as the ones involved in several activities of daily living, this can be explained by an additional ventilatory and postural load on the thoracic complex [7,8] and by the simultaneity of afferent and efferent muscle stimuli, with consequent respiratory muscle dyssynchrony during the execution of the tasks and consequent modification of the breathing pattern with implications for performance fatigability and upper-limb kinematics and movement [9,10].

Particularly in older individuals, fatigue can be a crucial factor in the performance of everyday activities [11]. Even in the absence of disability, aging causes disturbances in the biophysical characteristics of the muscle, but also due to neural factors [12]. Therefore, aged skeletal muscle is expected to become slower and weaker, and reveal a powerful decrease in the efficiency of voluntary contractions while also being less stable in the course of the efficiency of isometric contractions, especially at low force rates [13].

Performance fatigability, depicted as the “decline in an objective measure of physical performance over a discrete period” [14] is modulated by the contractile capacity of the muscles and the ability of the central nervous system to fulfill task requirements [15]. Physical tasks can be used to measure performance fatigability [16], where result variables can be the period that a task is able to be sustained (time-to-task failure—TTF), the change of pace in muscular activation, energy production, and other physiological settings [14]. Task demands, such as contraction strength, speed, balance, support for the fatigued limb, and the physiological features of the population (aging and sex) influence performance tiredness and the factors involved [17].

Since performance fatigability may also be revealed as decreased movement accuracy [18,19,20], impaired proprioception acuity [21], and decreased cocontraction during precision movements [18,22,23], the biomechanical approach can be used to identify alterations that occur in motion patterns all the time [24], namely those which are fatigue related.

Up to now, the study of kinematic changes caused by fatigue has involved optoelectronic or equivalent motion-capture systems. However, inertial motion units (IMUs) are nonintrusive and portable devices that allow kinematic assessment by blending data from a 3D gyroscope accelerometer, a 3D accelerometer, and a 3D magnetometer, and combining them with a fusion algorithm (e.g., Kalman filter) [25]. They also allow the acquisition of long-term data from real-time applications and in daily-life environments [25,26,27,28]. IMUs are becoming a common choice for experts conducting clinical tests in various rehabilitative settings [29], especially due to their portability, cost, and accessibility compared to other biomechanical analysis systems [27,30].

According to recent studies, IMU sensor systems accurately measure motor function and supply useful data concerning motor elements that promote assignment performance such as movement precision, smoothness, and accuracy [31], even in single IMU systems [32], showing encouraging results with respect to their reliability and intersystem agreement [33,34], especially in temporal parameters during activity [24] with higher validity for simple tasks [35].

Research on the application of IMUs for upper-limb movement analysis has grown in recent years, though their application is still at an early development stage and is especially targeted to the validation of specific protocols and proof-of-concept systems [26,34,36] and to movement analysis and characterization in specific clinical conditions [31,37]. As far as we are aware, there are only a few studies that have used IMUs to assess performance fatigability directly or indirectly during upper-limb [38,39] tasks, and none, during isometric tasks performed by elderly populations.

Understanding the position-sustained isometric task effects on upper-limb kinetics and performance fatigability is an important step to predict fatigue early based on movement kinetics assessed by an IMU, reducing the potentially harmful effects of the isometric task in these subjects, but also making it possible to prescribe more specific practices that increase the efficiency of the elderly in sports, recreational, or rehabilitation situations.

Consequently, the purpose of the present study was to understand how upper-limb kinetics behaves alongside activity time and how it relates to performance fatigability during an upper-limb position-sustained isometric task (ULPSIT). For that, we established two hypotheses: (1) During ULPSIT execution, there are modifications in upper-limb acceleration variation and (2) TTF is related to average acceleration variation.

## 2. Materials and Methods

A quasi-experimental prospective research design was implemented. The results of this research were reported following the CONSORT 2010 guidelines [40].

**Figure 1:**
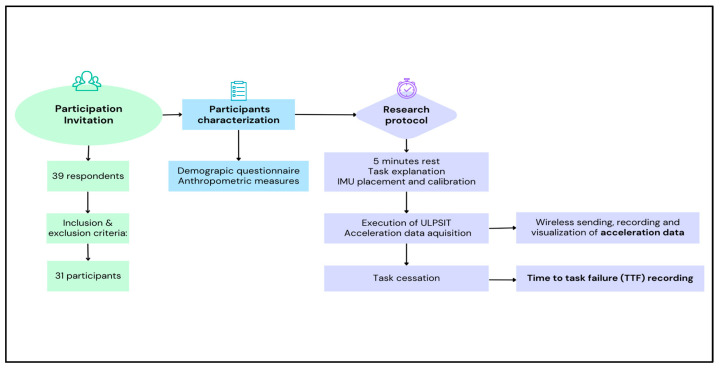
Sampling and research-procedures chart.

Sampling and research procedures are summarized in Figure 1.

### 2.1. Participants

The study included 31 elderly subjects (≥65 years old) (16 men and 15 women) screened from surrounding community institutions between October 2021 and February 2022.

Participants were informed about the aim of the research and read and signed the informed consent form voluntarily in accordance with the international principles of the Declaration of Helsinki [41]. This study was approved on 5 March 2021, by Escola Superior de Saúde da Cruz Vermelha Portuguesa—Lisboa Ethics Committee, Portugal (ESSCVP-EC_01/2021) and was registered at ClinicalTrials.gov (NCT04938791).

Participants were included if they were apparently healthy and more than 65 years old. The exclusion criteria were: (1) a cardiovascular and/or respiratory disease, hypertension, ischemic cardiopathy, or exercise disability, that can increase the risk of cardiovascular abnormalities performing the isometric activity; (2) cognitive or neurological imbalances that made subjects unable to understand or comply with the procedures of the study; (3) subjects with a body mass index (BMI) equal to or more than 40 to avoid the risk of cardiovascular diseases related to class III obesity in the course of physical activity; and finally, (4) neuromuscular disturbance that restricts the 90° flexion motion of the upper limb or the stance position.

G*Power 3.1.9.2 software was used to calculate the sample size (G*Power ©; University of Düsseldorf, Düsseldorf, Germany). To analyze the differences before and after performing the task, we used a pre–post study that used the variable of time-to-task failure point (TTF) throughout the upper-limb isometric task, which found a significant correlation in performance fatigability (TTF) (*p* < 0.01) [42]. Thus, to achieve a statistical confidence of 95%, with a 2-tailed hypothesis test and a large effect size of 0.90, an α-error of 0.05, and a power of analysis of 0.80 (β error = 20%) were chosen. The result achieved was 18 subjects. Keeping in mind the possibility of loss to follow up, 39 subjects were recruited.

From the 39 respondents recruited for eligibility, 7 subjects were excluded for meeting exclusion criteria (3 subjects for meeting exclusion criteria 1, and 4 subjects for meeting exclusion criteria 4), and 32 intentionally agreed to participate. However, one participant was excluded since the sensor crashed during activity, resulting in a sample of 31 participants composed of 15 women and 16 men, age range between 65 and 85 years old (age: 72.61 ± 5.23 years; weight: 73.18 ± 13.01 kg; height: 1.60 ± 0.08 m; BMI: 28.71 ± 4.66 kg·m^2^).

### 2.2. Inertial Measurement Unit (IMU) and Time-to-Task Failure (TTF)

The 3D acceleration of the upper limb was recorded through one IMU module (MTw Awinda, Xsens Technologies B.V., Enschede, The Netherlands). The orientation of the MTw is computed by Xsens Kalman Filter for a 3 degrees-of-freedom (3DoF) orientation for human motion (XKF3hm). XKF3hm is an algorithm that fuses 3D inertial data and 3D magnetometer data to optimally estimate 3D orientation with respect to an earth-fixed coordinate frame. MTw units run an improved signal-processing raw data and include patented StrapDown Integration (SDI) algorithms to dispatch the data wirelessly, through an Awinda Station (Xsens Technologies B.V, Enschede, The Netherlands), to a recording PC at a 100 Hz sampling rate, while keeping the precision of sampling at a higher rate (e.g., >1 kHz) [43]. Data visualization and recording were performed with MT Manager software, v. 4.4.0 (Xsens, Enschede, The Netherlands).

The time-to-task failure (TTF) in seconds was used as a performance fatigability outcome.

### 2.3. Research Procedure

The research was performed in the CrossLab, Health Research Lab at Escola Superior de Saúde da Cruz Vermelha Portuguesa, Lisboa in Portugal in a room with a constant temperature of 23 °C.

All the subjects followed the same overall protocol, and all data were collected by an expert researcher with more than 10 years of experience. All the subjects were instructed to avoid drinking caffeine beverages 2 h before the investigation.

Initially, the subjects were weighed and measured and then were invited to fill out a brief characterization survey. After completing the questionnaire, subjects took a rest for five minutes in a comfortable chair to relax their feet on the floor (resting position). Last-minute recommendations were made about the task they had to do after this period and the IMU was positioned.

**Figure 2:**
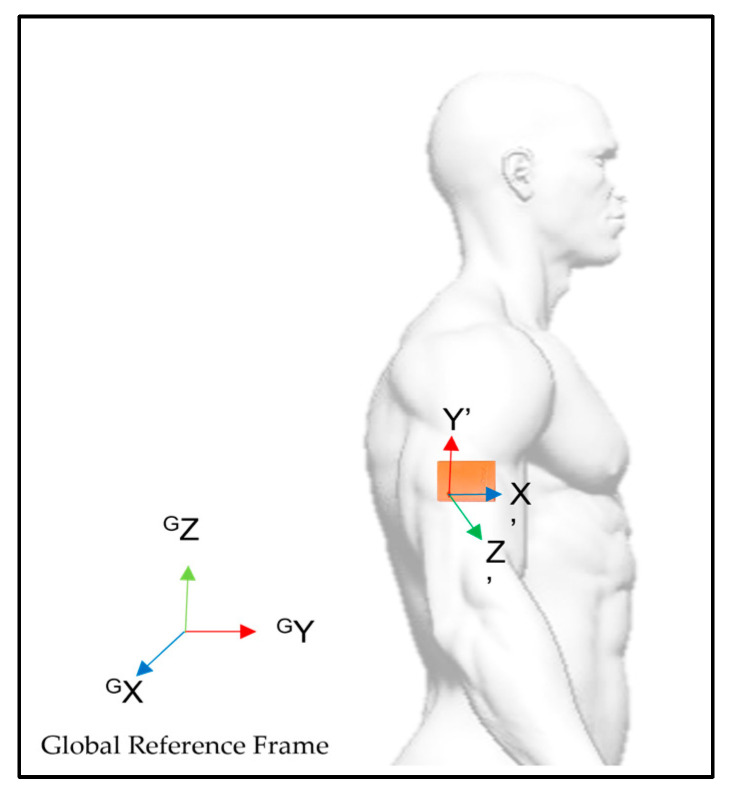
IMU placement and reference coordinate system before alignment reset.

The IMU was placed on the external side of the humerus of the dominant arm so that its reference coordinate system had the X-axis pointing forward, the Y-axis pointing upwards and the Z-axis pointing laterally, and perpendicularly to the sagittal plane (Figure 2). Double-sided tape was used to reduce soft-tissue artifacts. The IMU calibration procedure was conducted, aligning the IMU’s local coordinate system with the global reference coordinate system, with participants sitting in an N-pose facing forward for the measurement setting. In this way, the local coordinate system assumes the same orientation and alignment as the global coordinate system, allowing all the participants to have the same motion orientation and alignment, facilitating data analysis [43,44].

That said, the IMU motion came to be depicted in the global system of coordinates (x-y-z = north-west-up) so that the X-axis points forward, the Y-axis points laterally and the Z-axis points upward. Whenever there was upper-limb flexion, an increase of the X-axis value and a decrease of the Z-axis value was observed, and, on the contrary, a decrease of the X-axis value and an increase of the Z-axis value was observed during the extension motion. When sensor displacement occurred in the transversal plane it produced an increase in the Y-axis value if the displacement was medial (horizontal adduction), or a decrease, if the displacement was lateral (horizontal abduction).

Subjects were then asked to complete the task—the flexion of the upper arm until 90º with the hands facing each other and keeping in the same posture for as long as they could. Subjects were instructed to maintain trunk, neck, and head posture. Acceleration data were recorded during all of the activity periods until the failure point.

Subsequently, after the task was finished, subjects were asked to recover the rest posture and total activity time until the task failure was recorded.

### 2.4. Data Processing and Statistical Analysis

Acceleration data were extracted from the IMU, in the anterior–posterior axis (X-axis), medial–lateral axis (Y-axis), and vertical axis (Z-axis). Due to the heterogeneity in the activity duration of the subjects, it was necessary to standardize the time of data to compare them. Thus, ten cutoffs were established corresponding to the TTF deciles, from the beginning (baseline) through to the end of the task (100% TTF) in 10% interval cutoffs [45,46].

For each cutoff, the average acceleration was calculated. The baseline cutoff represents the data average of the first 15 s of activity after each participant had reached 90° upper-limb flexion.

The average acceleration variation ratio was also computed for each cutoff by dividing each participant’s cutoff value, in each axis, by their baseline value. So, the acceleration variation ratio reflects the proportion of change of acceleration toward baseline values in each cutoff. Cutoff values equal to 1 mean no modification in relation to baseline values and higher or less than 1 mean that there was, respectively, an increase or decrease in the average acceleration in relation to the baseline. Similar data processing has been used by other authors [29].

The activity time (TTF) and sociodemographic characteristics of the subjects were reported as mean, standard deviation (SD), and minimum and maximum values.

Acceleration variations alongside activity time were characterized through temporal features (as mean, standard deviation (SD), and minimum and maximum values) since, in static actions, the temporal features have a higher recognition rate [47].

The Shapiro–Wilk test was employed to analyze the variables with a normal distribution (*p* > 0.05). The demographic and anthropometric characteristics and Z-axis variables followed a normal distribution. According to this, Student’s *T*-test for independent samples was used to analyze the presence of significant differences among means of sexes; the Student’s T-test for paired samples was used to analyze significant differences between the means of two situations; Levene’s test was used to test the balance of variances. For non-normally distributed variables (TTF, X- and Y-axes), nonparametric tests were used: the Mann–Whitney U test for independent samples to compare variable differences between sexes; and the Wilcoxon signed-rank test to compare variable differences between different moments. Spearman’s correlation coefficient was calculated to measure the strength and direction of association between all variables. The level of significance was set at *p* < 0.05 with a CI of 95% for the statistical tests.

Statistical analyses were performed using SPSS statistical software, version 28.0 for Windows (IBM Company, Armonk, NY, USA).

## 3. Results

### 3.1. Sample Demographic and Anthropometric Characteristics

A sample of 31 subjects (16 men and 15 women) with ages between 65 and 85 years old (mean 72.61 ± 5.23 years), and mean body mass indexes (BMI) of 28.71 kg/m^2^ (±4.66 kg/m^2^) finished the research course. Participants’ demographic and anthropometric characteristics are detailed in Table 1.

All participants were right-handed. There were no statistically significant differences in age or BMI between the men and women. Nevertheless, the weight and height variables showed significant differences.

### 3.2. Acceleration Behavior of the Upper Limb during Isometric Activity

**Figure 3:**
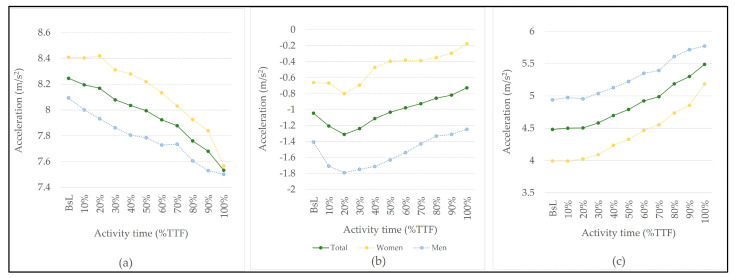
Average acceleration variation (raw data) in the total sample, women, and men: (**a**) in X–axis; (**b**) in Y–axis; and (**c**) in Z–axis alongside activity time.

The average acceleration showed different behavior depending on the axis of movement (Figure 3 and Table 2), with a progressive decrease on the X-axis, and an increase on the Z-axis. Regarding the Y-axis, there was an oscillation of acceleration throughout the activity, with the beginning of the activity (up to 20% of TTF) being characterized by a decrease in acceleration and then an increase until the end of the activity. Sex-related significant differences were only found in the Z-axis for the baseline and 10%TTF.

A standard deviation analysis shows an increasing spread of X-axis and Z-axis values alongside activity time.

The acceleration difference in relation to the baseline values (Table 3) was significant at 30%TTF and at 50%TTF for the X- and Z- axes respectively, and no significant difference in relation to the baseline values was identified for the Y-axis.

**Figure 4:**
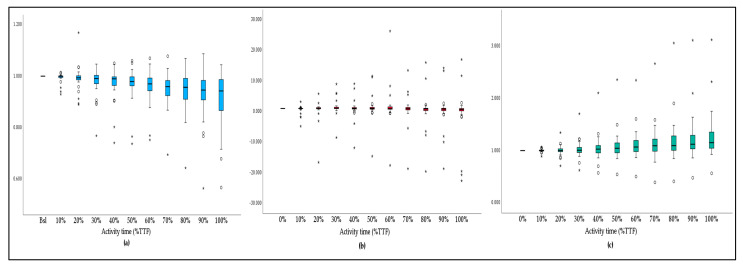
Acceleration ratio variation in (**a**) the X-axis; (**b**) the Y-axis; and (**c**) the Z-axis alongside the activity time. Values more than three IQR´s from the end of the box are labeled as extreme cases and denoted with an asterisk (*). Values more than 1.5 IQR´s but less than 3 IQR´s from the end of the box are labeled as outliers (^o^).

The difference relating to baseline values in the X- and Z- axes is evident when looking at the acceleration variation ratio alongside the activity-time graphics (Figure 4). In both cases, an increasing interquartile range (IQR) is also evident, showing an increase in the acceleration-ratio variability along with the activity execution. The Y-axis graphic shows irregular acceleration-ratio behavior, with a greater number of extreme cases, compared to the other axes, and an increasing IQR until the 70%TTF cutoff, followed by a decrease in the same parameter till the end of the activity.

**Figure 5:**
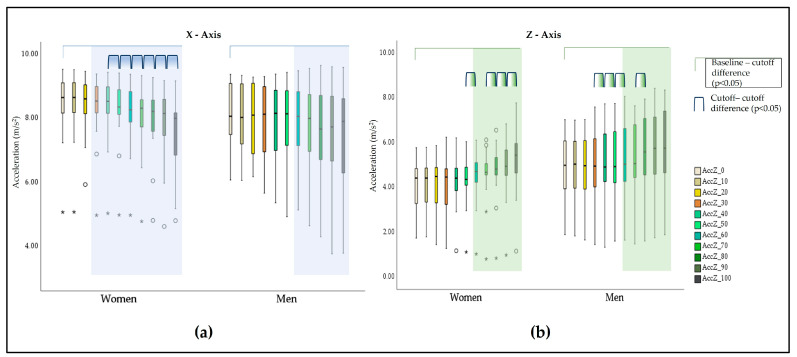
Sex-related differences in relation to baseline and between cutoffs in the X-axis (**a**)and the Z-axis (**b**). Values more than three IQR´s from the end of the box are labeled as extreme cases and denoted with an asterisk (*). Values more than 1.5 IQR´s but less than 3 IQR´s from the end of the box are labeled as outliers (^o^).

A sex influence was detected especially in the X-axis, with significant differences (*p* < 0.05) occurring earlier in the women (30%TTF, *p* = 0.041) than in the men (60%TTF, *p* = 0.039). In the Z-axis, significant differences occurred at 60%TTF, in both sexes (Table 3 and Figure 5).

Between consecutive cutoffs (Table 4), significant differences started between the 30–40%TTF cutoffs on the Z-axis (*p* = 0.007) with a moderate to large effect size and, between the 50–60%TTF cutoffs on the X-axis (*p* = 0.005) with a small to moderate effect size.

When looking for sex-related differences (Table 4 and Figure 5), we noticed that differences between cutoffs in the X-axis were significant only in the women and in the 40–50% and subsequent cutoffs. In the Z-axis, differences were found for the women in the second half of the activity, starting from the 50–60% cutoff, though in the men, significant differences were found from the 30–40% cutoff to the 50–60% and in the 70–80% cutoff.

The Y-axis showed no significant differences among baseline and across activity values or between consecutive cutoffs, except for the 40–50% cutoff where significant differences were identified with a small effect size. No influence of sex was identified.

A negative correlation was verified between the acceleration of the X- and Z-axes in all cutoffs. However, no significant correlation was detected between the Y-axis and the other axes (Table 5).

### 3.3. Time-to-task Failure

The average TTF was 474.52 s (Table 6). Sex analyses showed significant differences between the men and women, with the men having a longer activity time. In both cases, a high range of values and SD can be observed.

### 3.4. Acceleration Variation and TTF

When investigating the existence of relations between acceleration and the time spent performing the task (TTF), no correlation was detected for the total sample. However, a sex analysis revealed a positive correlation in the men between TTF and the X-axis in the 10% to 60% cutoff and a negative correlation between TTF and the Y-axis in the 10% and 30% cutoff (Table 7). No correlation was found for the women.

## 4. Discussion

Fatigability and the use of IMUs are becoming trends as clinical outcomes for measuring motor function and functional declines, though according to our understanding, there is no evidence that explores the use of IMUs and fatigability as outcomes in position-maintained isometric exercises of the upper limbs in the elderly.

This study aimed to understand how upper-limb kinetics behaves alongside activity time during a position-sustained isometric task and how it relates to performance fatigability. As hypothesized, there were kinematic changes in the acceleration behavior alongside the activity time in an ULPSIT, with differences between the sexes, especially in the second period of the exercise, and related to performance (TTF) in the men.

Despite participants being asked to perform a position-sustained isometric task (keeping the upper limb in the same position for as long as they could), it was possible to identify significant changes in average acceleration in the X- and Z-axes alongside activity time, indicative of motion of the upper limb in the sagittal plane and the shoulder extension direction, since the X-axis values decreased and the Z-axis values increased during activity time.

It is known that muscle fatigue leads to altered motor recruitment, and increased variability of force and movement [20,48,49], and that these changes are often associated with muscle fatigue [38] or the compensatory strategies that occur in the presence of fatigue, which gradually changed along the task time with the aim of relieving the effects of fatigue and maintaining performance for the maximum time possible [50,51].

Our acceleration data show movement in the sagittal plane which is in compliance with the findings of other authors for several types of upper-limb tasks (from simple to performance-based tasks) where the application of fatigue protocols produced kinematic changes and compensatory strategies involving the shoulder joint, which appears to prioritize performance rather than movement acuity.

In these studies, a reduction in the glenohumeral flexion, [19,51], an increase in the shoulder horizontal-abduction joint angles [46], changes in the trunk range of motion [19,46,51], and a combination of individual variations in the scapular kinematics to maintain an elevated shoulder position were observed, but also a decrease in movement accuracy during the task execution [20,52], with higher variability in movement trajectories in different axes [46,51].

The increasing variability in movement trajectories is also in accordance with our findings since an increasing acceleration variation was found that can be noted through standard deviation behavior, and in acceleration-variation ratio representation.

In the Y-axis, no significant statistical differences relating to baseline values or between cutoffs were detected, and neither was a significant correlation with other axes detected. These findings may be associated with the high variability in acceleration behavior among participants as shown in the acceleration variation ratio representation. However, data visualization of acceleration on this axis showed a tendency to increase acceleration alongside activity time in the transversal plane, which is compatible with a movement tendency to horizontal adduction.

The increase in movement variability that may occur as neuromuscular fatigue progresses, involves time-course changes in interjoint and intermuscular coordination and leads to a lack of force control [52], and a rise in movement complexity [46,48].

This can be related to the changes in muscle recruitment [49] with a modification of muscle synergies, since the role of a fatigued muscle within a muscle synergy structure may change, producing adaptations in the recruitment of the remaining muscles in the synergy structure to compensate [53,54], but also in the cocontraction (agonist–antagonist) of the muscles around the shoulder joint, since the decrease of muscular cocontraction negatively influences movement endpoint accuracy and increases endpoint movement variability [18].

So, the central nervous system (CNS) tries to compensate for the internal distress by centrally rearranging its motor schemes to maintain optimal task performance [49], though higher variability in movement trajectories in different axes is expected [46,51], until task failure occurs, especially because the nervous system fails to keep up enough muscular activation [55].

In this study, sex-related differences were also found relating to average acceleration behavior in the X- and Z-axes. In the X-axis, acceleration variation became significant in the women at 30% of activity time and maintained a significant variation between cutoffs in all of the second halves of the activity; in the men, acceleration variation became significant only at 60% of activity time without significant differences between cutoffs.

In the Z-axis, acceleration variation became significant at the same timepoint for the men and women, though differences seem to have a major influence in the phase of activity in which variation of acceleration is more significant: in the middle third of the activity in the men and the second part of the activity in the women.

The sex-related findings suggest that movement adjustments occurred in different ways. In the women, changes in relation to baseline values occurred earlier, and in the X-axis, which may indicate greater differences than the ones identified for the men in the Z-axis, in the same activity phase, but between consecutive cutoffs, without a significant difference in relation to baseline values.

These findings may also suggest that the X-axis is more sensitive to changes in acceleration in relation to baseline values and the Z-axis is more sensitive to changes in acceleration between cutoffs since both axes are involved in movements in the sagittal plane.

As seen before, significant acceleration variability suggests greater variability in movement, which may indicate movement adjustments and an increase in movement variability, possibly associated with fatigue and caused by changes in muscle recruitment. Thus, the data obtained indicate that fatigue appeared in the women and the men in the same phase of activity, though with differences in the magnitude of adjustments.

It was possible to determine a positive correlation between the average acceleration in the X-axis and the TFF, between 10% and 60% TFF, despite the variation in acceleration not being significant until 60% TTF. This may indicate that, in the men, minor adjustment movements occur in an early phase, and were sufficient to increase activity time and, consequently, performance. This is supported by the fact that the men showed a higher TFF compared to the women, suggesting greater fatigability of performance in the women than in the men.

Our findings were not in accordance with the evidence from previous research that has shown that usually, men were more fatigable than women when performing isometric contractions at low to moderate intensities [56,57,58,59] and that older subjects were less fatigable than young subjects for upper- and lower-limb muscles for an isometric-contraction fatiguing task at the same relative intensity [52,60].

However, it is well known that these discrepancies are task specific [57,61], rely on the details and demands of the task [17], and are associated with anatomical, biomechanical, and physiological factors [17,42,56,62,63].

This may justify our findings since factors that may have contributed to fatigue in this task may not be critical to another. Another possibility is related to the low number of subjects in each sex group which may have conditioned the results obtained. Therefore, future studies should address a larger number of participants of both sexes in order to verify (or not) these results.

This research provides findings about acceleration behavior during an upper-limb position-sustained isometric task in a sample of elderly people, which is an important step to predict fatigue early and reduce the potentially harmful effects of upper-limb isometric tasks in this population.

However, its findings should be read in the context of some limitations that are acknowledged. First, the randomization sampling procedure should be carried out with a greater sample to reduce sample bias and clarify some of the results obtained. Second, since TTF is dependent on the will of the participants to maintain (or not) the task, and is related to perceived fatigability [14,42], TTF may be underestimated for some participants. So, in future studies, it would be important to add other performance and perceived fatigability outcomes to check the perceptual influence on performance. Third, IMU-related limitations such as soft-tissue artifacts, magnetic disturbance [30], and lack of standardization on sensor placement [34] can negatively influence data accuracy. To reduce these limitations, IMU placement was standardized and applied always by the same investigator, using double-sided tape to reduce soft-tissue artifacts. The IMU calibration procedure was carried out, and laboratory conditions were maintained in order to reduce magnetic oscillations. Other IMU-related limitations, such as drift and the gimbal-lock phenomenon, were avoided by the task position that was used and also since no angular features were used. However, in the future, it would be interesting to use an IMU system to measure the orientation behavior of the upper limb and other kinematic features during ULPSIT.

This research has some strengths. Our sample was composed of men and women, giving the chance to explore sex’s influence on the results. We studied the average acceleration data extracted from the IMU, with a simple data-processing procedure, which means that this study protocol can be easily used and replicated by professionals without high competence in data processing and in several environments, especially since today there are commercially available instruments, such as smartwatches and smartphones, that also include accelerometers.

In elderly people, it is essential to detect high-fatigability status early and to develop specific measurements and activities that can respond to the necessity of this population, delaying frailty and fatigue appearance, and increasing their self efficacy and functional performance.

Considering that we found that the average acceleration variations that occur during a ULPSIT are compatible with fatigue-related movement adjustments, upper-limb acceleration measured through a single IMU can be a useful and easy strategy to identify fatigue early. However, studies are needed that relate these results with perceived fatigability and neuromuscular measurements simultaneously.

## 5. Conclusions

The results of this research showed that a simple kinematic protocol based on one IMU module is capable of detecting changes in acceleration behavior during an upper-limb position-sustained isometric task, indicative of movement of the upper limb in the sagittal plane (in the direction of shoulder extension), especially in the second part of the task, and increasing variability in movement alongside activity time.

The results also showed that average acceleration variation differed between men and women, with the women having major adjustments sooner than the men, which can indicate that fatigue appeared first in the women. This is supported by the fact that, in this group, there is a lower TFF compared to the men, suggesting greater performance fatigability in the women. TTF was positively related to average acceleration only in the men, where minor adjustment movements in the Z-axis occurred in an early phase, though apparently sufficient to increase activity time and, consequently, performance.

These findings suggest that a simple IMU module can be used as a valuable instrument in rehabilitation and sports environments to early identify fatigue-related changes in the acceleration of the upper limb, be able to modify strategies, and enhance the performance of the elderly. However, some limitations have been identified related to sampling and the influence of perceived fatigability, so future studies should address the use of larger and random samples and the use of perceived fatigability outcomes and other performance fatigability outcomes. In addition, the use of an IMU system, with more modules, may allow the measurement of other kinematic features during ULSPIT, such as orientation through pitch, raw, and yaw angles).

## Figures and Tables

**Table 1:** Sociodemographic and anthropometric characteristics.

Sample Characteristics	Total Sample Mean ± SD (Range)	Women Mean ± SD (Range)	Men Mean ± SD (Range)	*p* Value ^1^	Effect Size Cohen’s d
Age (years)	72.61 ± 5.23	72.27 ± 6.05	72.94 ± 4.49	0.727	0.126
	(65–85)	(65–85)	(65–82)		
Weight (kg)	73.18 ± 13.01	67.57 ± 12.37	78.44 ± 11.61	0.009	0.907
	(48–100)	(48–86.5)	(57.90–100)		
Height (m)	1.60 ± 0.08	1.54 ± 0.43	1.65 ± 0.06	<0.001	2.116
	(1.48–1.76)	(1.48–1.64)	(1.57–1.76)		
BMI (kg/m^2^)	28.71 ± 4.66	28.69 ± 5.30	28.74 ± 4.15	0.490	0.695
	(21.57–37.94)	(21.57–37.94)	(23.42–35.61)		

Abbreviations: BMI, body mass index; SD, standard deviation. *p* < 0.05 value was considered statistically significant. ^1^ Independent samples Student’s *t*-test.

**Table 2:** Acceleration of raw data alongside activity time.

Activity Time (TTF)	X-axis					Y-axis					Z-axis				
	Total Mean ± SD (Range)	Women Mean ± SD (Range)	Men Mean ± SD (Range)	*p* Value ^1^	Effect Size *r*	Total Mean ± SD (Range)	Women Mean ± SD (Range)	Men Mean ± SD (Range)	*p* value ^1^	Effect Size *r*	Total Mean ± SD (Range)	Women Mean ± SD (Range)	Men Mean ± SD (Range)	*p* Value ^2^	Effect Size Cohen´s d
Baseline	−8.25 ± 1.04 (5.07–9.53)	8.41 ± 1.11 (9.53–5.07)	8.09 ± 0.97 (9.37–6.08)	0.264	0.206	−1.05 ± 2.47 (−4.14–6.74)	−0.66 ± 2.94 (6.74–−3.62)	−1.40 ± 1.96 (−4.14–1.97)	0.740	0.064	4.48 ± 1.35 (1.74–7.02)	3.99 ± 1.18 (5.77–1.74)	4.94 ± 1.37 (7.02–1.90)	0.048	0.742
10%	8.20 ± 1.07 (5.08–9.52)	8.40 ± 1.10 (9.52–5.08)	8.00 ± 1.04 (9.34–6.06)	0.202	0.234	−1.20 ± 2.48 (−4.72–6.74)	−0.67 ± 2.95 (6.74–−3.61)	−1.71 ± 1.90 (−4.72–0.96)	0.599	0.099	4.50 ± 1.37 (1.80–7.00)	3.99 ± 1.19 (5.79–1.80)	4.98 ± 1.40 (7.00–1.84)	0.045	0.754
20%	8.17 ± 1.05 (5.93–9.47)	8.42 ± 0.93 (9.47–5.93)	7.93 ± 1.12 (9.29–6.18)	0.202	0.234	−1.31 ± 2.48 (−5.48–5.83)	−0.80 ± 2.82 (5.83–−3.64)	−1.79 ± 2.07 (−5.48–1.27)	0.654	0.085	4.51 ± 1.41 (1.45–7.03)	4.02 ± 1.20 (5.87–1.45)	4.96 ± 1.47 (7.03–1.66)	0.064	0.691
30%	8.08 ± 1.20 (4.97–9.38)	8.31 ± 1.14 (9.38–4.97)	7.86 ± 1.24 (9.30–5.67)	0.338	0.177	−1.24 ± 2.58 (−5.41–6.85)	−0.70 ± 3.04 (6.85–−3.60)	−1.75 ± 2.04 (−5.41–1.39)	0.626	0.092	4.58 ± 1.47 (1.28–7.59)	4.09 ± 1.23 (6.24–1.28)	5.04 ± 1.57 (7.59–1.45)	0.073	0.669
40%	8.04 ± 1.23 (5.04–9.44)	8.28 ± 1.10 (9.44–5.04)	7.81 ± 1.33 (9.38–5.36)	0.423	0.149	−1.11 ± 2.56 (−5.31–6.80)	−0.47 ± 3.00 (6.80–−3.56)	−1.71 ± 1.97 (−5.31–1.38)	0.520	0.121	4.70 ± 1.47 (1.17–7.73)	4.24 ± 1.18 (6.21 – 1.17)	5.13 ± 1.61 (7.73–1.33)	0.091	0.629
50%	7.99 ± 1.24 (4.93–9.43)	8.22 ± 1.10 (9.41–4.98)	7.78 ± 1.35 (9.43–4.93)	0.520	0.121	−1.03 ± 2.55 (−5.09–6.92)	−0.40 ± 3.06 (6.92–−3.62)	−1.63 ± 1.85 (−5.09–1.13)	0.446	0.142	4.79 ± 1.45 (1.12–7.75)	4.33 ± 1.16 (6.04–1.12)	5.22 ± 1.59 (7.75–1.61)	0.085	0.640
60%	7.92 ± 1.25 (4.98–9.49)	8.13 ± 1.09 (9.37–4.98)	7.73 ± 1.38 (9.49–5.14)	0.572	0.107	−0.98 ± 2.49 (−4.87–6.80)	−0.38 ± 3.06 (6.80–−3.61)	−1.54 ± 1.72 (−4.87–0.81)	0.545	0.114	4.92 ± 1.51 (1.03–8.07)	4.47 ± 1.23 (6.11–1.03)	5.35 ± 1.66 (8.07–1.65)	0.105	0.602
70%	7.88 ± 1.21 (4.65–9.56)	8.03 ± 1.13 (9.33–4.78)	7.73 ± 1.31 (9.56–4.65)	0.599	0.099	−0.93 ± 2.53 (−4.53–7.14)	−0.39 ± 3.16 (7.14–−3.70)	−1.43 ± 1.72 (−4.53–0.75)	0.682	0.078	4.99 ± 1.51 (0.80–7.64)	4.56 ± 1.30 (6.12–0.80)	5.39 ± 1.61 (7.64–1.48)	0.123	0.571
80%	7.76 ± 1.27 (4.30–9.65)	7.93 ± 1.17 (9.27–4.81)	7.61 ± 1.38 (9.65–4.30)	0.599	0.099	−0.86 ± 2.45 (−4.13–7.01)	−0.35 ± 3.10 (7.10–−3.71)	−1.33 ± 1.57 (−4.13–0.89)	0.861	0.036	5.19 ± 1.58 (0.84–7.94)	4.74 ± 1.37 (6.56–0.84)	5.61 ± 1.68 (7.94–1.61)	0.125	0.568
90%	7.68 ± 1.36 (3.77–9.61)	7.84 ± 1.23 (9.18–4.62)	7.53 ± 1.50 (9.61–3.77)	0.682	0.078	−0.82 ± 2.39 (−3.85–7.25)	−0.29 ± 3.11 (7.25–−3.85)	−1.31 ± 1.36 (−3.77–0.74)	0.922	0.021	5.30 ± 1.63 (0.98–8.42)	4.86 ± 1.40 (6.84–0.98)	5.72 ± 1.75 (8.42–1.75)	0.143	0.541
100%	7.53 ± 1.37 (3.79–9.59)	7.57 ± 1.28 (9.17–4.81)	7.50 ± 1.49 (9.59–3.79)	0.984	0.007	−0.73 ± 2.28 (−3.83–6.68)	−0.17 ± 2.98 (6.68–−3.83)	−1.25 ± 1.21 (−3.54–0.65)	0.654	0.085	5.49 ± 1.64 (1.16–8.35)	5.19 ± 1.54 (7.77–1.16)	5.77 ± 1.73 (8.35–1.89)	0.328	0.357

In all analyses, *p* < 0.05 was considered statistically significant. ^1^ Mann-Whitney U test; ^2^ Independent samples Student’s *t*-test. Abbreviations: TTF, time to task failure; SD, standard deviation.

**Table 3:** X-, Y-, and Z-axes baseline—cutoff differences alongside activity time.

	X-Axis						Y-Axis						Z-Axis					
	Total Sample *p* Value ^1^	Effect Size *r*	Women *p* Value ^1^	Effect Size *r*	Men *p* Value ^1^	Effect Size *r*	Total Sample *p* Value ^1^	Effect Size *r*	Women *p* Value ^1^	Effect Size *r*	Men *p* Value ^1^	Effect Size *r*	Total Sample *p* Value ^2^	Effect Size Cohen’s d	Women *p* Value ^2^	Effect Size Cohen’s d	Men *p* Value ^2^	Effect Size Cohen’s d
Baseline-10%TTF	0.100	0.209	0.691	0.073	0.079	0.311	0.183	0.169	0.820	0.041	0.148	0.256	0.416	0.148	0.968	0.011	0.378	0.227
Baseline-20%TTF	0.068	0.231	0.061	0.342	0.179	0.238	0.117	0.199	0.609	0.093	0.121	0.274	0.679	0.075	0.622	0.130	0.872	0.041
Baseline-30%TTF	0.018	0.301	0.041	0.373	0.098	0.293	0.389	0.109	0.865	0.031	0.234	0.210	0.252	0.210	0.399	0.225	0.453	0.192
Baseline-40%TTF	0.006	0.348	0.015	0.446	0.070	0.320	0.969	0.005	0.281	0.197	0.352	0.165	0.062	0.349	0.171	0.373	0.229	0.313
Baseline-50%TTF	0.003	0.383	0.005	0.508	0.070	0.320	0.597	0.067	0.307	0.187	0.717	0.064	0.020	0.441	0.111	0.439	0.105	0.432
Baseline-60%TTF	<0.001	0.431	0.004	0.529	0.039	0.366	0.570	0.072	0.334	0.176	0.959	0.009	0.003	0.580	0.039	0.589	0.043	0.552
Baseline-70%TTF	<0.001	0.465	0.003	0.550	0.034	0.375	0.505	0.085	0.427	0.145	0.796	0.046	0.002	0.606	0.047	0.561	0.017	0.673
Baseline-80%TTF	<0.001	0.478	0.002	0.570	0.023	0.402	0.410	0.105	0.460	0.135	0.569	0.101	<0.001	0.704	0.025	0.649	0.008	0.757
Baseline-90%TTF	<0.001	0.500	0.001	0.591	0.020	0.411	0.367	0.114	0.460	0.135	0.501	0.119	<0.001	0.746	0.014	0.726	0.009	0.746
Baseline-100%TTF	<0.001	0.498	0.001	0.591	0.044	0.357	0.299	0.132	0.427	0.145	0.501	0.119	<0.001	0.851	0.002	0.954	0.010	0.737

Abbreviations: TTF, time-to-task failure. *p* < 0.05 value was considered statistically significant. ^1^ Wilcoxon signed-rank test; ^2^ Paired samples Student’s *t*-test.

**Table 4:** X-, Y-, and Z-axes consecutive cutoff differences alongside the activity time.

	X-Axis						Y-Axis						Z-Axis					
	Total Sample *p* Value ^1^	Effect Size *r*	Women *p* Value ^1^	Effect Size *r*	Men *p* Value ^1^	Effect Size *r*	Total Sample *p* Value ^1^	Effect Size *r*	Women *p* Value ^1^	Effect Size *r*	Men *p* Value ^1^	Effect Size *r*	Total Sample *p* Value^2^	Effect Size Cohen´s d	Women *p* Value ^2^	Effect Size Cohen´s d	Men *p* Value ^2^	Effect Size Cohen´s d
10–20%TTF	0.117	0.199	0.156	0.259	0.278	0.192	0.256	0.144	0.496	0.125	0.379	0.155	0.902	0.022	0.546	0.160	0.721	0.091
20–30%TTF	0.085	0.219	0.088	0.311	0.438	0.137	0.272	0.139	0.460	0.135	0.438	0.137	0.082	0.323	0.303	0.276	0.172	0.359
30–40%TTF	0.256	0.144	0.394	0.156	0.379	0.155	0.153	0.182	0.211	0.228	0.535	0.110	0.007	0.524	0.066	0.515	0.033	0.587
40–50%TTF	0.104	0.207	0.031	0.394	0.836	0.037	0.033	0.271	0.140	0.270	0.134	0.265	0.004	0.558	0.051	0.552	0.045	0.546
50–60%TTF	0.005	0.358	0.002	0.570	0.255	0.201	0.108	0.204	0.691	0.073	0.063	0.329	<0.001	0.917	0.001	1.053	0.006	0.794
60–70%TTF	0.030	0.276	0.017	0.435	0.501	0.119	0.117	0.199	0.776	0.052	0.070	0.320	0.239	0.216	0.164	0.380	0.637	0.120
70–80%TTF	0.002	0.403	0.009	0.477	0.063	0.329	0.126	0.194	0.570	0.104	0.179	0.238	<0.001	0.806	0.007	0.809	0.006	0.790
80–90%TTF	0.075	0.226	0.041	0.373	0.605	0.091	0.308	0.129	0.281	0.197	0.796	0.046	0.007	0.523	0.036	0.598	0.091	0.451
90–100%TTF	0.040	0.261	0.020	0.425	0.679	0.073	0.367	0.114	0.394	0.156	0.605	0.091	0.010	0.493	0.014	0.728	0.382	0.225

Abbreviations: TTF, time-to-task failure. *p* < 0.05 value was considered statistically significant. ^1^ Wilcoxon signed-rank test; ^2^ Paired samples Student’s *t*-test.

**Table 5:** Correlation between the acceleration of the X- and Z-axes.

%TTF	Total Sample	Women	Men
Baseline	−0.851 ** 95% CI [−0.926, −0.712]	−0.821 ** 95% CI [−0.938, −0.533]	−0.909 ** 95% CI [−0.968, −0.753]
10%	−0.853 ** 95% CI [−0.927, −0.715]	−0.811 ** 95% CI [−0.934, −0.512]	−0.876 ** 95% CI [−0.956, −0.673]
20%	−0.869 ** 95% CI [−0.935, −0.744]	−0.854 ** 95% CI [−0.950, −0.608]	−0.874 ** 95% CI [−0.955, −0.668]
30%	−0.882 ** 95% CI [−0.941, −0.768]	−0.914 ** 95% CI [−0.971, −0.756]	−0.888 ** 95% CI [−0.960, −0.701]
40%	−0.877 ** 95% CI [−0.939, −0.759]	−0.871 ** 95% CI [−0.956, −0.648]	−0.915 ** 95% CI [−0.970, −0.768]
50%	−0.876 ** 95% CI [−0.938, −0.757]	−0.850 ** 95% CI [−0.949, −0.599]	−0.918 ** 95% CI [−0.971, −0.775]
60%	−0.874 ** 95% CI [−0.937, −0.753]	−0.846 ** 95% CI [−0.946, −0.583]	−0.929 ** 95% CI [−0.975, −0.804]
70%	−0.825 ** 95% CI [−0.912, −0.666]	−0.736 ** 95% CI [−0.906, −0.360]	−0.941 ** 95% CI [−0.979, −0.835]
80%	−0.864 ** 95% CI [−0.932, −0.735]	−0.782 ** 95% CI [−0.924, −0.451]	−0.941 ** 95% CI [−0.979, −0.835]
90%	−0.848 ** 95% CI [−0.924, −0.706]	−0.664 ** 95% CI [−0.877, −0.230]	−0.971 ** 95% CI [−0.990, −0.917]
100%	−0.882 ** 95% CI [−0.941, −0.768]	−0.739 ** 95% CI [−0.907, −0.365]	−0.979 ** 95% CI [−0.992, −0.939]

Spearman’s correlation coefficient (ρ). ** Statistically significant correlation *p* < 0.01 (2 tail). Abbreviations: TTF, time-to-task failure.

**Table 6:** TTF characterization.

	Total Sample Mean ± SD (Range)	Women Mean ± SD (Range)	Men Mean ± SD (Range)	*p* Value ^1^	Effect Size Cohen’s d
TTF (sec)	474.52 ± 301.41	369.60 ± 228.79	572.88 ± 333.95	0.037	0.706
	(128–1443)	(128-1012)	(286–1443)		

Abbreviations: TTF, time-to-task failure; SD, standard deviation., *p* < 0.05 value was considered statistically significant. ^1^ Independent samples Mann–Whitney U test.

**Table 7:** Correlation between the average acceleration and TTF.

Axis Cut-off	Men
X 10%	0.524 * 95% CI [0.022, 0.815]
X 20%	0.726 ** 95% CI [0.347, 0.902]
X 30%	0.656 ** 95% CI [0.222, 0.873]
X 40%	0.547 * 95% CI [0.054, 0.825]
X 50%	0.635 ** 95% CI [0.188, 0.864]
X 60%	0.503 * 95% CI [−0.006, 0.805]
Y 10%	−0.509 * 95% CI [−0.808, −0.001]
Y 30%	−0.603 * 95% CI [−0.850, −0.137]

Abbreviations: TTF, time-to-task failure. Spearman’s correlation coefficient (ρ). * Statistically significant correlation *p* < 0.05 value; ** Statistically significant correlation *p* < 0.01 value (2 tail).

## Data Availability

The dataset supporting the conclusions of this article is available upon request to h.smigueis@udc.es in the Research, Health and Podiatry Group, Department of Health Sciences, Faculty of Nursing and Podiatry.
